# Rb-82 generator yield

**DOI:** 10.1007/s12350-020-02287-x

**Published:** 2020-09-07

**Authors:** Adrian Nunn, Peter Oehlberg

**Affiliations:** 1 MI-Pharma Consulting, LLC, Lambertville, NJ USA; 2 Bracco Diagnostics Inc, Monroe Township, NJ USA

We read with interest the article by Ahmadi et al.^1^ reporting the results of an investigation led by the inventors and developers of RUBY-FILL®, one of the two marketed ^82^Sr/ ^82^Rb generators being compared.

We are concerned that the authors incorrectly state that the CardioGen-82® infusion system does not report the integrated total ^82^Rb activity as part of daily quality control (QC) procedures, but rather the dose calibrator maximum value. They also provide no support for their statement that neither the Patient Dose nor the End-of-Infusion activity values appear to be accurate in the CardioGen-82®daily QC breakthrough elution reports. Moreover, based on the use of a correction factor of 1.314 applied to the dose calibrator maximum values, they suggest that the RUBY-FILL® generator yield is ~ 7% higher than that of CardioGen-82®.

The CardioGen-82® system does provide an integrated dose for each infusion, the Patient Dose, as the authors ultimately acknowledge. For CardioGen-82®, the average difference between the dose calibrator maximum value and the Patient Dose for daily QC is actually a factor of 1.426, not the 1.314 the authors derived—a difference of ~ 8%.^2^ Thus, if the true correction factor with the dose calibrator maximum values or the actual integrated calibration doses are used, CardioGen-82® yields are similar to RUBY-FILL ® yields.

However, the concept of a yield or elution efficiency is arbitrary when the product has such a short half-life as ^82^Rb, because the 'yield' is dependent on the elution conditions. As the authors note, these are quite different both during daily QC and during normal use for the two systems. What matters is whether the user can get the desired doses over the lifetime of the generator. In this regard, the maximum dose available is constrained for both generators by the half-life of the ^82^Sr.
